# A Changing Landscape of Physician Quality Reporting: Analysis of Patients’ Online Ratings of Their Physicians Over a 5-Year Period

**DOI:** 10.2196/jmir.2003

**Published:** 2012-02-24

**Authors:** Guodong Gordon Gao, Jeffrey S McCullough, Ritu Agarwal, Ashish K Jha

**Affiliations:** ^1^Center for Health Information and Decision SystemsRobert H Smith School of BusinessUniversity of MarylandCollege Park, MDUnited States; ^2^Division of Health Policy & ManagementSchool of Public HealthUniversity of MinnesotaMinneapolis, MNUnited States; ^3^Department of Health Policy and ManagementHarvard School of Public HealthHarvard UniversityBoston, MAUnited States

**Keywords:** Physician quality, online reviews, patient empowerment, quality transparency, public reporting

## Abstract

**Background:**

Americans increasingly post and consult online physician rankings, yet we know little about this new phenomenon of public physician quality reporting. Physicians worry these rankings will become an outlet for disgruntled patients.

**Objective:**

To describe trends in patients’ online ratings over time, across specialties, to identify what physician characteristics influence online ratings, and to examine how the value of ratings reflects physician quality.

**Methods:**

We used data from RateMDs.com, which included over 386,000 national ratings from 2005 to 2010 and provided insight into the evolution of patients’ online ratings. We obtained physician demographic data from the US Department of Health and Human Services’ Area Resource File. Finally, we matched patients’ ratings with physician-level data from the Virginia Medical Board and examined the probability of being rated and resultant rating levels.

**Results:**

We estimate that 1 in 6 practicing US physicians received an online review by January 2010. Obstetrician/gynecologists were twice as likely to be rated (*P* < .001) as other physicians. Online reviews were generally quite positive (mean 3.93 on a scale of 1 to 5). Based on the Virginia physician population, long-time graduates were more likely to be rated, while physicians who graduated in recent years received higher average ratings (*P* < .001). Patients gave slightly higher ratings to board-certified physicians (*P* = .04), those who graduated from highly rated medical schools (*P* = .002), and those without malpractice claims (*P* = .1).

**Conclusion:**

Online physician rating is rapidly growing in popularity and becoming commonplace with no evidence that they are dominated by disgruntled patients. There exist statistically significant correlations between the value of ratings and physician experience, board certification, education, and malpractice claims, suggesting a positive correlation between online ratings and physician quality. However, the magnitude is small. The average number of ratings per physician is still low, and most rating variation reflects evaluations of punctuality and staff. Understanding whether they truly reflect better care and how they are used will be critically important.

## Introduction

There is broad consensus among policy makers and consumer groups that greater transparency in health care will improve the quality and costs of care delivered [1]. The US federal government, through the auspices of the Centers for Medicare & Medicaid Services (CMS), has led several transparency initiatives, including publicly reporting individual hospital performance on process measures and standard metrics of patient experience [2,3]. However, such efforts, when directed toward individual physicians, have been far more controversial and generally lagged behind reporting for other providers [4,5]. In the CMS’s recent physician reporting initiative, individual performance measures will not be available until 2013 [6]. Policy makers and physician groups worry about having an adequate sample size to create stable estimates and not penalizing physicians who care for sicker or disadvantaged patients [5,7–9]. While national efforts at physician performance reporting have progressed slowly, a new phenomenon has begun to fill the gap.

Internet-based consumer ratings of physicians have gained interest from the private sector and are seen by many as an extension of similar user-submitted rating services, such as those focused on restaurants, hotels, books, or plumbers [10]. Advocates argue that such rating systems will provide consumers much-needed information about physician quality (at least from the consumer experience perspective) [11]. Making greater use of patient feedback is also consistent with patient empowerment, a goal set by the Affordable Care Act [12]. Critics worry that the Internet rating sites will be a forum for disgruntled patients to vent frustration over minor shortcomings, and that a small number of such ratings might tarnish physicians’ reputation [13,14]. Professional societies such as the American Medical Association and even some state governments have expressed concerns about these rating programs [15,16]. Since most rating websites do not require the authentication of raters, online ratings may be subject to manipulation.

Despite these concerns, there is reason to believe that these ratings will become commonplace. Patients frequently turn to online sources for health information. While only 19% of American adults indicated that they were very likely to seek health information over the Internet in 2001 [4,8], 59% of American adults searched for health information online in 2010 [17]. Furthermore, 16% of Internet users have consulted online ratings or reviews of doctors or other providers [17,18]. This growth suggests that there is tremendous potential for these ratings to affect physician livelihood and patient behavior, but only limited studies exist [19–22]. To shed light on the growing phenomenon of online physician ratings, we used data from a major user-submitted physician review rating site to answer four questions. What proportion of US physicians have received online ratings and how has this changed over time? What types of physicians are likely to be rated? What kinds of ratings do physicians receive? And finally, are certain characteristics of physicians, such as board certification status or history of malpractice payouts, correlated with higher ratings?

## Methods

### Data

We developed a new dataset incorporating patients’ online ratings and physician characteristics, captured from RateMDs.com. Although the number of websites that offer physician ratings has increased substantially in recent years, we focused on RateMDs.com for the following reasons. First, among the rating sites, RateMDs.com has the largest number of user-submitted reviews with narratives by a large margin, based on a recent study by Lagu et al [21] and a website ranking service [23]. Second, all the physician reviews on RateMDs.com are submitted by users voluntarily, rather than populated by surveys. There are no incentives for users to submit ratings, and the ratings are publicly available and free to use. Third, RateMDs.com started in 2004 and is one of the earliest physician review websites in the United States, while most other major competitors began rating services only after 2008. Given its early entry, RateMDs.com data enabled us to construct a 6-year period from the website’s inception through January 2010 to derive insights into the historical growth trend. We retrieved all the available doctor ratings on RateMDs.com up to January 2010.

We used the Area Resource File to examine the number and distribution of US physicians, including the number of active nonfederal physicians, by specialty and geographic area. Area Resource File is a national health resource information database published by the US Department of Health and Human Services. We linked the 2009 Area Resource File data to physician ratings, by state, to better understand the growth and prevalence of patients’ ratings and variations in ratings across different specialties.

To determine, in a more granular fashion, factors associated with patient rating decisions, we obtained data from the Virginia Board of Medicine. These data provide detailed information on all licensed physicians practicing in the Commonwealth of Virginia. We chose Virginia because it is a relatively large state that provides relatively detailed data on licensed physicians. We matched the ratings database to the Virginia data on the basis of name, address, and specialty. We found that the distribution of ratings across and within specialties in Virginia was very similar to that seen in the national population.

### Measures

RateMDs.com’s physician ratings have four domains: staff, punctuality, helpfulness, and knowledge. Patients rate physicians in each of the domains on a scale of 1 to 5, with 1 being the lowest and 5, highest. The website automatically generates an overall physician quality measure based on the average of helpfulness and knowledge ratings. While we examined all four domains, we focused on the overall quality rating as our primary outcome.

We also captured data from the website about each physician’s specialty. We grouped the 97 specialties identified into five major types of physicians: primary care, medical specialists, surgeons including surgical specialists, obstetricians and gynecologists, and other specialists (such as radiologists and pathologists). One additional rationale for separating obstetricians and gynecologists is their unique patient population (women mostly between 20 and 40 years old). We identified the location of each practice and the date of each rating. We aggregated the location data into the four broad census regions: Northeast, South, Midwest, and West.

To obtain more granular details about specific physician characteristics and their association with both the likelihood of being rated and the ratings themselves, we linked the online ratings to physicians’ board certification records for the Commonwealth of Virginia. Our data from the Virginia board had 18,174 physicians, a census of Virginia’s actively practicing licensed physicians as of January 2010. We matched all Virginia physicians listed on the RateMDs.com database to the Virginia licensing board data. Based on this match, we calculated that about 22% of the physician entries were duplicates due to misspelling or variation of physicians’ names, which is typical for user-submitted ratings. Therefore, in all subsequent national data, we applied a correction factor (reducing the number of physicians by 22%) to calculate national statistics. We also found that patients accurately identified their physician's specialty greater than 95% of the time, suggesting that our national data are likely accurate in physician specialty designations.

We used the medical board data to determine specialty, graduation year, medical school, and malpractice claim history for each physician. We grouped the specialties into the same five major categories used for the patient rating data. After identifying the year of medical school graduation for each physician, we divided graduation years into four categories: before 1980, 1980–1989, 1990–1999, and 2000–2009. Third, we identified the medical school attended by each physician. We used a binary variable to indicate whether the medical school was ranked among the top 50 schools by *U* *.* *S* *.* *News & World Report*. Finally, we determined whether each physician had paid out any malpractice claims in the past 10 years of practice.

### Analysis

We determined the total number of ratings over time for each of the five specialty categories and each geographical region. We also determined the average number of ratings per physician. Next, we examined the distribution of ratings and determined the average physician rating for each of the five specialty categories.

Among physicians practicing in Virginia, we determined the percentage who had been rated in the demographic categories of specialty, graduation year, board certification, ranking of medical school, and malpractice claim history. We used both bivariate and multivariable regression techniques to examine each of the individual characteristics and their association with the likelihood of being rated as well as the rating itself.

## Results

Through January 2010, we found that more than 112,000 physicians had been rated through RateMDs.com. Compared with the national physician composition, rated physicians were more likely than the national population to be obstetrician/gynecologists and more likely to be a medical specialist (see Table 1). Male physicians were somewhat more likely to be rated (83,043/112,024, 74.13%) than would be expected based on their national composition (503,529/703,223, 71.60%), and physicians in the South were slightly more likely to be rated (39,684/112,024, 35.43%) than their national composition (233,332/703,223, 33.18%).

**Table 1:** Comparison of the physicians rated in RateMDs.com versus the national physician population.

		Rated physicians (n = 112,024)		National physicians^a^ (n = 703,223)	
**Specialty**					
	Primary care	45,552	40.66%	280,273	39.86%
	Medical specialties	17,754	15.85%	72,073	10.25%
	Surgeon/surgical specialties	22,657	20.23%	113,011	16.07%
	Obstetrics/gynecology	12,978	11.59%	40,013	5.69%
	Other specialties	13,083	11.68%	197,853	28.14%
**Gender**					
	Male	83,043	74.13%	503,529	71.60%
	Female	28,981	25.87%	199,694	28.40%
**Region**					
	Northeast	25,663	22.91%	168,600	23.98%
	South	39,684	35.42%	233,332	33.18%
	West	23,742	21.19%	153,552	21.84%
	Midwest	22,935	20.47%	147,739	21.01%

^a^ Based on 2007 Area Resource File patient-care physician data.

### Physician Rating Trends

**Figure 1:**
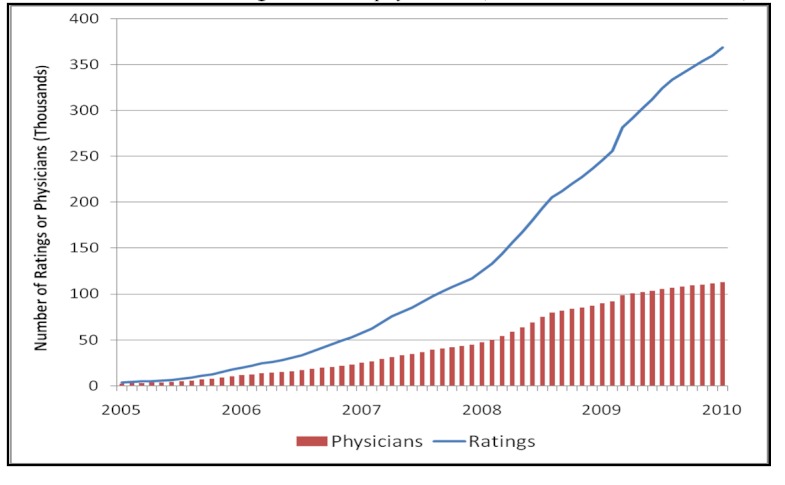
Cumulative number of ratings and rated physicians (based on data from RateMDs.com).

From the inception of RateMDs.com in 2004, there has been strong enthusiasm from voluntary users. By January 31, 2010, after excluding non-US and nonphysician practitioners, there were a total of 368,559 physician ratings (Figure 1), a 100-fold increase in the preceding 5 years. The number of individual physicians who had at least one online rating grew commensurately from 2475 physicians in January 2005 to 112,024 by January 2010, covering about 16% of all practicing US physicians.

**Figure 2:**
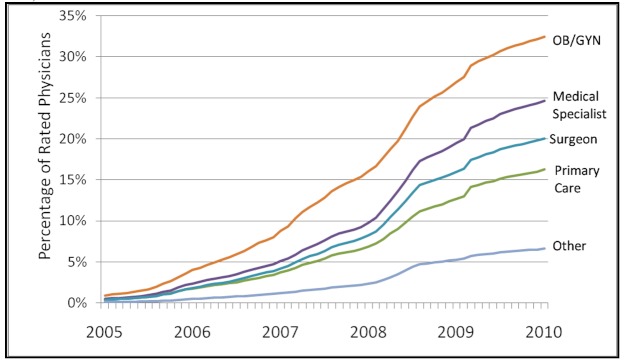
Percentage of US physicians rated in each specialty (based on data from RateMDs.com and the US Department of Health and Human Services’ Area Resource File). OB/GYN = obstetrician/gynecologists.

The likelihood of being rated varied widely across specialties as of January 2010: while 32.43% (12,978/40,013) of all obstetrician/gynecologists had been rated, approximately 24.63% (17,754/72,073) of medical specialists, 20% (22,657/113,011) of surgeons, and 16.25% (45,552/280,273) of primary care physicians had received a rating. Only 6.61% (13,083/197,853) of physicians classified as other specialists (such as radiologists, pathologists, and anesthesiologists) had been rated (Figure 2). This pattern is consistent across the regions.

The average number of ratings per physician in January 2010 was 3.2, and nearly half of the physicians had only a single rating. However, the number of physicians with five or more ratings rose rapidly from less than 1% in 2005 to 12.50% (14,003/112,024) in 2010.

### Distribution of Ratings

**Figure 3:**
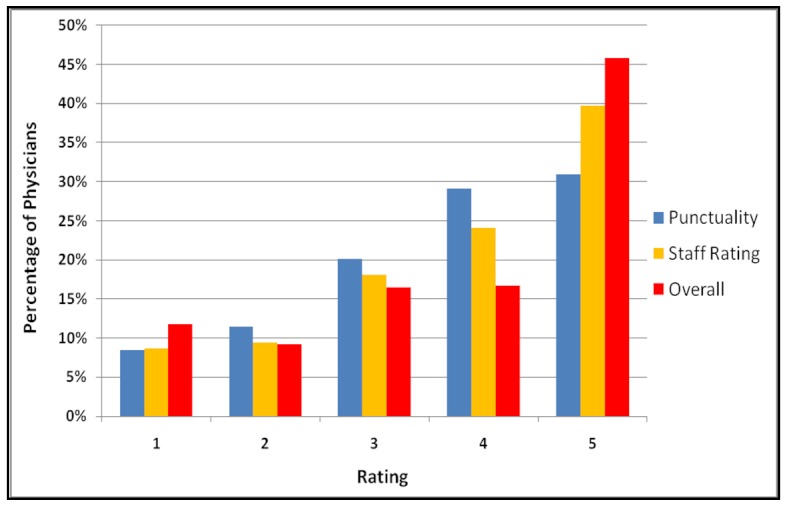
Distribution of quality ratings across physicians.

The average quality rating, which is based on the physician’s helpfulness and knowledge, was high (3.93 out of 5), with 45.80% (51,307/112,024) of physicians receiving a 5 out of 5, while only 11.76% (13,174/112,024) of ratings were below 2 (Figure 3). Ratings on the two other dimensions (quality of staff and punctuality of physician), while still generally positive, were somewhat lower: only 30.88% (34,593/112,024) of ratings for punctuality were a 5 out of 5, while 39.68% (44,451/112,024) of the ratings for staff helpfulness were a 5 out of 5 (Figure 3). The correlation between physician quality rating and staff rating was .73 (*P* < .001), and the correlation between quality and punctuality was .68 (*P* < .001), consistent with a previous finding [22].

Across specialties, we found that the mean quality ratings were similar for physicians in primary care (4.02), medical specialties (3.96), surgeon and surgical specialties (3.89), and obstetrician/gynecologists physicians (4.01). Physicians listed within the group of other specialties had lower ratings (3.59), a difference that was statistically significant (data not shown). Finally, male physicians had, on average, a somewhat higher rating than female physicians (3.95 vs 3.89, *P* < .001).

### Virginia Physicians’ Likelihood of Being Rated

Of the 18,174 physicians in Virginia, 3164, or 1 out of every 6, had received at least one rating, which was similar to the national rate. These Virginia physicians had a total of 10,534 ratings by January 31, 2010, with an average rating of 3.3 per physician, also consistent with the national data. Much like in the national data, obstetrician/gynecologists were far more likely than others to be rated (see Table 2). Younger physicians (those graduating from medical school after 2000) were much less likely to be rated. We found that physicians who were board certified and those who had at least one paid malpractice claim were more likely to be rated, although the difference based on malpractice claim did not reach statistical significance (*P* = .12). Graduates of more highly ranked medical schools were rated with nearly the same frequency as graduates of lower-ranked medical schools. When we used multivariable models to examine the association of these factors with the likelihood of being rated, we found nearly identical results (see Multimedia Appendix 1, Table 1).

**Table 2:** Virginia physicians by quality rating status.

		Virginia physicians	Unadjusted rate of being rated (%)	*P* value^a^
Number of physicians		18,174	17	
**Specialty**				<.001
	Primary care	6540	19	
	Medical specialties	2806	20	
	Surgeon/surgical specialties	2751	22	
	Obstetrics/gynecology	1145	37	
	Other specialties	4932	7	
**Graduation year**				<.001
	Before 1980	5142	17	
	1980–1989	5276	20	
	1990–1999	5184	20	
	2000–2009	2572	9	
**Board certification**				<.001
	Board certified	15,057	19	
	Not board certified	3117	10	
**Medical school ranking**				.42
	Ranked top 50^b^	4962	18	
	Ranked below top 50	13,212	17	
**Malpractice claims**				.12
	No malpractice claims	16,886	17	
	At least one malpractice claim	1288	23	

^a^ Based on joint χ^2^ test for subgroup differences with other controls.

^b^ Based on 2008 *U* *.* *S* *.* *News & World Report* ranking.

We found modest effects of specialty on the quality rating that physicians received. Consistent with our national data, Virginia physicians classified as practicing other specialties had moderately lower ratings (3.63) than other physician specialty categories (Table 3). The differences between the other specialists, such as primary care physicians (4.04), medical specialists (3.95), surgeons (3.90), and obstetrician/gynecologists (4.04) were small and not significant.

Younger physicians—those who had graduated from medical school after 2000—had significantly higher ratings than older physicians. While there were small differences across the different age cohorts (3.85 for physicians graduating before 1980, 3.95 for those graduating in the 1980s, and 3.99 for those graduating in the 1990s), the youngest cohort had an average rating of 4.22 (*P* < .001 for differences across groups). Board-certified physicians had somewhat higher ratings than physicians who were not board certified (3.96 vs 3.86, *P* = .04). Similarly, physicians graduating from a top-50 medical school had somewhat higher ratings than other physicians (4.08 vs 3.91, *P* = .002). Physicians with no history of paying malpractice claims were rated somewhat higher than physicians who had at least one malpractice claim, although this difference did not reach statistical significance (*P* = .1). Once again, we found very similar results in our multivariable models (see Multimedia Appendix 1, Table 2).

**Table 3:** Virginia physicians by the value of quality rating.

		Unadjusted average rating	*P* value^a^
All rated physicians		3.95	
**Specialty**			<.001
	Primary care	4.04	
	Medical specialties	3.95	
	Surgeon/surgical specialties	3.90	
	Obstetrics/gynecology	4.04	
	Other specialties	3.63	
**Graduation year**			<.001
	Before 1980	3.85	
	1980–1989	3.95	
	1990–1999	3.99	
	2000–2009	4.22	
**Board certification**			.04
	Board certified	3.96	
	Not board certified	3.86	
**Medical school ranking in primary care**			.002
	Ranked top 50^b^	4.08	
	Ranked below top 50	3.91	
**Malpractice claims**			.1
	No malpractice claims	3.97	
	At least one malpractice claim	3.82	

^a^ Based on joint *F* test for subgroup differences with other controls.

^b^ Based on 2008 *U* *.* *S* *.* *News & World Report* ranking.

## Discussion

We examined physician ratings on a major user-submitted physician review website in the United States. We found dramatic growth in the number of physicians being rated (1 in 6 practicing doctors in the nation in January 2010). This trend was widespread across the nation, and its penetration differed based on physician specialty. Not surprisingly, physicians with less direct patient contact (such as pathologists or radiologists) were infrequently rated. On the other hand, obstetrician/gynecologists were far more likely to be rated than others with nearly 1 in 3 such physicians now having an online rating at RateMDs.com. This could be attributed to the younger and female patient population, who are more likely to be active Internet users. However, the difference between specialties in the propensity to be rated deserves future study. We found that ratings were generally quite positive; however, patients were more critical in their ratings of staff and punctuality.

Although some physicians are concerned that online ratings will become a channel for disgruntled patients to vent their complaints [13,14], our findings suggest that this is not the most common reason patients use the rating system. In fact, given that nearly half the physicians received a perfect 5 out of 5, online ratings appear to be driven by patients who are delighted with their physicians. Conversely, only 12% of ratings were below 2. These findings should allay concerns that online rating sites disproportionately attract dissatisfied patients; however, the prevalence of high ratings may reflect ratings selection regarding which physicians are rated.

One major concern with the physician ratings is the small sample sizes. The average number of ratings per physician is only three, and approximately half of all physicians were rated by only one patient. This low density of ratings makes physicians’ average ratings vulnerable to large swings from the input of a single patient or manipulation from providers.

By linking the ratings with Virginia Board of Medicine licensing data, we found that board-certified physicians as well as those who attended higher-ranked schools had better ratings, although the differences were small. There are several potential explanations. First, it is possible that patients were aware of their physicians’ school rankings or their board certification status and were, therefore, biased toward being more favorable. Alternatively, board-certified physicians from highly ranked schools might care for a patient population more positively predisposed to ranking their physicians highly. While these are all plausible, it is also possible that patients are adept at identifying high-quality physicians, and their rankings reflect the generally better care that these physicians might provide. Certainly, prior studies have found that patients rate their experiences more highly in hospitals that have higher-quality performance [3,24], and our findings might reflect a similar phenomenon in the ambulatory setting. The finding that physicians with a history of paid malpractice claims had slightly lower ratings further supports this explanation. By linking individual physician characteristics to their online ratings, this study provides the first statistical evidence on how online ratings by patients are associated with physician quality. Further work is needed to discern whether these ratings are correlated with actual clinical outcome.

The notion that patients will use the Internet for medical information should come as no surprise. Some studies have found that 61% of US adults have looked online for health information, and among them 24% have consulted rankings or reviews online of doctors or other providers [17]. Given that patients place substantial trust in online health information (with some studies suggesting, surprisingly, that patients may place more trust in Internet-based health information than opinions of friends or coworkers [18]), the use of these kinds of data for choosing providers will likely become commonplace.

Indeed, the potential use of these data by consumers is a concern that has led some organizations to speak out against these rating programs, worried that negative ratings might harm physician practice volume and could affect physician livelihood [25]. Others, such as the National Health Service in the United Kingdom, have taken a different tack: starting October 2009, they are encouraging patients to rate their general practitioners through a National Health Service-run website [26]. Lagu and Lindenauer recently called on the CMS to put the public back in public reporting by allowing consumers to report their experiences on Hospital Compare and other government-run websites that feature provider quality performance [11].

A limitation of our work is that we aggregated data from a single website. Although it is ranked highly among physician review websites [23], in recent years, several other websites including healthgrades.com, vitals.com, yelp, and Angie’s List have been offering physician ratings and are growing in popularity. Lagu et al identified 33 websites that provide physician ratings [21]. However, most of the major competitors did not start physician ratings until very recently, and some report patient survey results rather than patient-initiated reviews. Nevertheless, the values we generated for the number of physicians who have an online reputation almost certainly understate the phenomenon. Unfortunately, aggregating data across multiple websites is impractical given the potential number of practicing US physicians, the growing number of websites that offer the physician rating service, and incompatibility in rating methods and review collection approaches [20,22]. Our estimates therefore provide a lower bound of the magnitude of this important phenomenon. Future studies could examine patient choice of different rating websites.

Another limitation of our work is that our data are based on a limited number of ratings for physicians, although in aggregate, they reflect the current state of the ratings program. Further, given that the phenomenon of physician ratings is relatively new, we suspect that as more patients rate their physicians, the associations we found may change over time. Additionally, since the website could not verify the identities of the authors of reviews, it is possible that some ratings were subject to manipulation. Although the website has taken certain actions, including disallowing multiple ratings for a physician from the same computer, and removing self-promoting reviews once detected, the possibility of manipulation cannot be completely eliminated. Finally, our physician characteristics data came from a single state, and although the state appeared to be representative in the ways we could measure (ie, percentages of physicians rated, the rating scores themselves, etc), it is unclear whether the associations found using Virginia data generalize to the rest of the nation. We also lack finer measures of physician quality to be associated with the online ratings.

In conclusion, this study makes unique contributions to our understanding of online doctor ratings by examining its national growth trend, and by identifying influential factors such as specialty, board certification, education, and malpractice claims. We found that ratings were generally positive, and certain types of clinicians were both more likely to be rated and to be rated more highly. Whether the medical community and policy makers are supportive of this phenomenon or not, our findings suggest that user-generated reviews of providers are here to stay and likely to grow. We also found a weak correlation between the online ratings and physician quality. This ought to create greater impetus for policy makers to provide a context for user-generated data by speeding up current efforts to report physician quality scores online. The explosion of this information suggests that consumers are not only generating these data but likely also using it. Given the potential impact of this phenomenon, there is a great need to examine the information value and potential biases inherent in Web-based ratings, how they are being used, the impact they are having in physician choice (if any), and how to help patients make the best use of the online ratings to complement other physician performance information [27].

## Multimedia Appendix 1

Statistical appendix.

## Abbreviations

CMS: Centers for Medicare & Medicaid Services
