# Supplementary material for: A Changing Landscape of Physician Quality Reporting: Analysis of Patients’ Online Ratings of Their Physicians Over a 5-Year Period
Source: J Med Internet Res. 2012 Feb 24;14(1):e38. doi: 10.2196/jmir.2003 (PMC3374528; doi:10.2196/jmir.2003)
Supplement: Supplementary file 1 [file jmir_v14i1e38_app1.pdf]

A changing landscape of physician quality reporting: Analysis of patients' online ratings of their physicians over a 5-year period

Appendix

We find that the likelihood of having online ratings is significantly associated with board certification, specific specialties, graduation years, and malpractice claims. The logistic regression is reported in Appendix Table 1. Being board certified increases the odds of being rated by 62%. Among specialties, obstetrician/gynecologists are most likely to be rated online, followed by surgical physicians and primary care physicians. In contrast, physicians in "other specialties" are the least likely to be rated online. With respect to year of graduation, physicians graduating between 1980 and 1989 are most likely to be rated online, followed by physicians who graduated between 1990 and 1999. Young physicians who graduate after year 2000 are much less likely to be rated. Physicians who have malpractice claims also have a higher chance of being rated online, with the odds increasing by 12%, but not statistically significant. The ranking of the medical school seems to have no influence on the odds ratio of being rated online.

We then examined the effect of these characteristics on average quality rating values using a multivariate OLS regression, as reported in Appendix Table 2. Board certified physicians and those graduating from top 50 medical schools received higher average ratings; with the magnitude of the difference being 0.16 points (about a 4% increase). Ratings were slightly lower for physicians with at least one malpractice claim, and the differences were marginally significant.

We found some significant differences across specialties. Ratings for physicians in primary care, medical specialties and obstetrician/gynecologists were not significantly different from one another on average. Surgeons' ratings were slightly lower than those of primary care physicians by 0.11 point, corresponding to a 3.5% reduction. The average rating for physicians in other specialties was 0.39 points lower than primary care physicians, representing approximately a 10% drop. Finally, we found that younger physicians had notably higher ratings. Their ratings were nearly 10% higher than those of older physicians.

Appendix Table 1: Adjusted odds ratios for being rated derived from a regression model

	**Odds Ratio (95% CI)**	**P-Value**
**Specialty**		
Primary care	1.00	-
Medical specialties	0.94 (0.84-1.06)	0.34
Surgeon/surgical specialties	1.07 (0.96-1.20)	0.23
OB/GYN	2.40 (2.09-2.76)	<0.001
Other specialties	0.28 (0.25-0.0.32)	<0.001
**Graduation year**		
Before 1980	1.00	-
1980-1989	1.22 (1.10-1.35)	<0.001
1990-1999	1.16 (1.04-1.28)	0.006

2000-2009	0.53 (0.45-0.63)	<0.001
**Board certification**		
Not Board certified	1.00	-
Board certified	1.62 (1.42-1.85)	<0.001
**Medical school ranking in primary care**		
Ranked top 50**a**	1.00	-
Ranked below top 50	0.96 (0.88-1.05)	0.42
**Malpractice claims**		
With no malpractice claims	1.00	-
With at least one malpractice claim	1.12 (0.97-1.29)	0.12

[a]based on 2008 US News and World Report ranking.

Appendix Table 2. OLS regression of the value of quality ratings on physician characteristics

	**Coefficient (95% CI)**	**P-Value**
**Specialtya**		
Medical specialties	-0.07 (-0.19-0.06)	0.279
Surgeon/surgical specialties	-0.11 (-0.24-0.01)	0.068
OB/GYN	0.01 (-0.13- 0.15)	0.875
Other specialties	-0.389 (-0.54 - -0.23)	<0.001
**Graduation yearb**		
1980-1989	0.09 (-0.02-0.21)	0.100
1990-1999	0.12 (0.00-0.23)	0.047
2000-2009	0.41 (0.22-0.61)	<0.001
**Board certified**	0.16 (0.01-0.31)	0.037
**Medical school ranking in top 50 in primary care**	0.16 (0.06-0.26)	0.002
**With at least one malpractice claim**	-0.13 (-0.28-0.02)	0.099

[a]Primary care physicians are the comparison group

[b]Before 1980 is the comparison group
